# Energy-adjusted dietary inflammatory index is associated with chronic kidney disease-associated pruritus in Hemodialysis patients: a cross-sectional study

**DOI:** 10.1186/s12882-025-04211-2

**Published:** 2025-07-01

**Authors:** Hosein Rostami, Marjan Delkhosh

**Affiliations:** 1 Health Research Center, Life Style Institute, Baqiyatallah University of Medical Sciences, Tehran, Iran; 2 Department of Community Health and Geriatric Nursing, Faculty of Nursing & Midwifery, Tehran University of Medical Sciences, Tehran, Iran

**Keywords:** Dietary inflammatory index, Pruritus, Hemodialysis patients, Cross-sectional study

## Abstract

**Background:**

Chronic kidney disease-associated pruritus (CKD-aP) is a common and bothersome symptom among hemodialysis (HD) patients. This study aimed to determine the association of Energy-adjusted dietary inflammatory index (E-DII) index with the risk and severity of CKD-aP in hemodialysis patients.

**Methods:**

This cross-sectional study was conducted on 200 HD patients. A valid 168-item semi-quantitative Food Frequency Questionnaire (FFQ) was used to assess the usual food intake and calculated E-DII. Yosipovitch Itch Questionnaire was used to assess CKD-aP. Based on the E- DII score, odds ratios, and 95% confidence intervals were calculated for the risk of CKD-aP. Generalized linear models (GLM) was used to determine the association between E-DII scores and CKD-aP score, and pruritus Visual Analogue Scale (VAS) score.

**Results:**

The continuous E-DII score was associated with an increased risk of CKD-aP in all three models: model 1, OR = 1.19 (95% CI: 1.02–1.40); model 2, OR = 1.29 (1.01–1.65); and model 3, OR = 1.41 (1.01–1.98). Moreover, linear regression analysis showed statistically significant associations (*P* < .05) between the E-DII score and both CKD-aP and VAS scores across all three models.

**Conclusions:**

This study provides evidence that higher consumption of a pro-inflammatory diet is associated with an increased risk of CKD-aP in HD patients. Future studies with prospective and interventional designs are required to clarify the association between this dietary index and CKD-aP in HD patients.

## Introduction

Chronic kidney disease-associated pruritus (CKD-aP) is a common and bothersome symptom among end-stage renal disease (ESRD) patients under hemodialysis (HD) treatment [1]. The pathophysiology of CKD-aP is still not clear. Factors such as inadequate dialysis, uremic toxins, secondary hyperparathyroidism, hyperphosphatemia, iron deficiency anemia, biocompatible membranes, heparin, and skin xerosis have been known as risk factors of CKD-aP [2]. Moreover, previous studies have shown that CKD-aP is associated with systemic inflammation. In HD patients, inflammatory markers such as C-reactive protein (hs-CRP), interleukin 2 (IL-2), and interleukin 31 (IL-31) are associated with CKD-aP [3–6]. These markers play pivotal roles in modulating neural and immune responses. The activation of inflammatory pathways can sensitize cutaneous sensory neurons, particularly unmyelinated C-fibers, which are primarily responsible for transmitting pruritic signals. This sensitization results in an enhanced perception of itch [7]. Additionally, systemic inflammation may compromise the integrity of the skin barrier, leading to increased transepidermal water loss and xerosis, thereby exacerbating CKD-aP [8–10].

Several studies have indicated that diet plays a crucial role in regulating low-grade chronic inflammation [11]. The energy-adjusted dietary inflammatory index (E-DII) has been developed to evaluate the inflammatory potential of diet and has been validated in more than 36 construct validations using various inflammatory biomarkers [12, 13]. Previous studies have demonstrated the direct correlation between DII scores and chronic diseases such as chronic kidney disease [14], obesity [15], diabetes mellitus [16], and cardiometabolic risk [17]. Furthermore, several cross-sectional studies have reported a positive correlation between DII scores and CRP levels in HD patients [18, 19].

To the best of our knowledge, no study has examined the association between E-DII and CKD-aP in HD patients. It was hypothesized that a diet with a higher inflammatory potential could be associated with CKD-aP in HD patients.

## Materials and methods

### Participants

This analytical cross-sectional study was conducted on 200 HD (HD) patients. The study participants were selected by clustered random sampling from government hospitals including Imam Khomeini, Gholestan, and Razi Hospitals in Ahvaz City, Iran. Inclusion criteria included: age over 18 years and receiving regular HD for more than 12 months. Exclusion criteria included: patients with enteral or parenteral feeding, cognitive or communication problems, severe neurological or mental disorders, active neoplastic disease, severe alcohol or drug addiction, major amputation (lower/upper extremities), diagnosis of cancer, acute or chronic pancreatitis, irritable bowel syndrome, prolonged gastrointestinal symptoms, acute or chronic pancreatitis, hepatic insufficiency, patients with immunoinflammatory diseases, such as those with systemic lupus erythematosus, and energy intake below 800 kcal or above 4200 kcal.

### Sample size

The G*Power 3.1.9.4 software was then used to calculate the minimum sample size required for the study. In this regard, the minimum sample size reached with statistics (i.e., significance = 0.05; power = 0.95, and effect size = 0.33) was 196, based on the CKD-aP Score calculated using the Yosipovitch Itch Questionnaire (YIQ) in a study conducted in Iran [20].

### Data analysis

Part of the demographic data was collected from the dialysis unit’s database, while the remaining demographic information was obtained through a questionnaire administered via interviews. A physical activity questionnaire (IPAQ) was used to evaluate the physical activity, and the results were expressed as metabolic equivalent hours per week (METs hr/wk) [21]. Anthropometric, biochemical, and dialysis treatment data were retrieved from the dialysis unit’s database for the same month. All laboratory biochemical measurements were conducted using identical methods in different laboratories. Serum creatinine was measured with a colorimetric assay, serum urea with a photometric assay, serum albumin using the Bromocresol green method, serum phosphorus with a photometric technique, serum calcium with a colorimetric method, and serum potassium using flame photometry. All biochemical tests were performed using commercial kits from Pars Azmoon (Tehran, Iran).

### Dietary assessment

Trained dietitians obtained information on the usual food intake of all participants using a valid and reliable 168-item semi-quantitative Food Frequency Questionnaire (FFQ) with standardized servings [22]. The participants were requested to provide information on the frequency of their consumption of each food item, indicating whether it was consumed daily, weekly, or monthly, over the preceding year. These reported frequencies were then converted into gram-weight equivalents. To determine the total energy and nutrient intake, Nutritionist IV software (the Hearst Corporation) was utilized, with adjustments made to accommodate Iranian food items.

### DII calculation

DII scores were calculated using the methodology provided by Shivappa et al. [12, 13]. The index is derived from an analysis of 1,943 articles published between 1950 and 2010 which examined the impact of 45 dietary parameters on inflammatory biomarkers. To compute the DII score, the dietary data of each study participant was initially linked to a comprehensive global database that provided robust estimates of mean intake and standard deviation (SD) for each of the 45 food parameters. In the present study, the DII score was computed for 32 food parameters from the original list of 45, including energy, protein, carbohydrates, fiber, total fat, cholesterol, saturated, monounsaturated, and polyunsaturated fatty acids, trans fats, omega-3 and omega-6 fatty acids, vitamins A, E, D, C, B1, B2, B3, B6, B12, beta carotene, folate, zinc, iron (Fe), selenium, magnesium, garlic, onion, caffeine, pepper, and green/black tea. The E-DII, designed to control for the effects of total energy intake, was calculated based on the published literature [23]. Firstly, all dietary intake values were converted to values per 1000 kcal. Secondly, this energy-adjusted dietary intake was standardized as a z-score. To obtain the z-score, the actual intake of each food parameter was subtracted from the standard mean global intake for that parameter, and the result was divided by its global SD. Thirdly, to minimize skewness, the z-score was converted to the centered proportion score by multiplying it by 2 and subtracting 1. Fourthly, to acquire the food parameter–specific DII score, the final value was multiplied by its respective overall food parameter score. Finally, the overall E-DII score for each participant was calculated by summing the E-DII scores for all food parameters. Higher E-DII scores indicate a higher pro-inflammatory potential in the diet, while more negative values suggest a more anti-inflammatory diet [12, 13]. The E-DII scores were also categorized into quartiles.

### Assessment of pruritus

The Yosipovitch Itch Questionnaire (YIQ) was developed by Gil Yosipovitch et al. (2001) based on the McGill Questionnaire and has been validated for assessing CKD-aP in previous studies [24]. The YIQ is a comprehensive tool designed to assess the severity and impact of CKD-aP on patients’ lives. This questionnaire evaluates the history of itching and anti-itch drug intake, the impact of CKD-aP on sleep, daily activities, quality of life, and emotional dimensions of itching. Additionally, the questionnaire assesses the severity of CKD-aP using the Visual Analogue Scale (VAS) [22]. The scoring system incorporates various question formats to capture different dimensions of the itch experience. Dichotomous questions (Yes/No) are scored with ‘Yes’ responses assigned a value of 1 and ‘No’ responses a value of 0. Frequency-based questions assess the occurrence of itch-related experiences and are scored as follows: ‘Never’ = 0, ‘Sometimes’ = 1, and ‘Always’ = 2. Questions regarding the frequency of itch episodes are scored based on their occurrence, with daily episodes receiving the highest score of 4 and monthly episodes the lowest score of 1. Similarly, questions about the duration of each itch episode are scored based on length, with episodes lasting several minutes receiving a score of 1 and those lasting several hours receiving a score of 4. The Visual Analog Scale (VAS) measures the intensity of itch, where patients rate their itch on a continuum from 0 to 10, with 0 representing ‘no itch’ and 10 denoting the ‘worst imaginable itch’. To obtain the total score, individual item scores are summed, providing an aggregate measure of itch severity and its impact. Higher total scores indicate more severe CKD-aP and a greater effect on the patient’s quality of life. Additionally, categorization of itch severity is performed based on VAS scores, with specific cut-off points defining mild, moderate, and severe CKD-aP. The VAS score is classified as mild [1–3], moderate [4–6], and severe (7 and above) [25].

### Statistical analysis

The statistical analysis was conducted using IBM SPSS Statistics software (Version 24) from IBM SPSS Statistics, Armonk, USA. A significance level of less than 0.05 was considered statistically significant. The normality of variables was assessed using the Kolmogorov-Smirnov test. To compare variables across E-DII quartiles, a one-way analysis of variance (ANOVA) with Least Significant Difference (LSD) post hoc analysis was employed for normally distributed data. For non-normally distributed data, the Kruskal-Wallis test was utilized, and for categorical variables, the Chi-square test was applied. In Generalized linear models (GLM), the relationship between continuous dependent variables (CKD-aP and VAS) and E-DII quartiles (as an independent variable) was examined in three models: Model 1 without any adjustment; Model 2 with adjustment for age, sex, BMI, physical activity, diabetes, hypertension, smoking, job, marital status, education, income status, and energy intake; and Model 3 with adjustment for model 2 in addition to inter-dialysis weight gain, dialysis vintage, dialysis time, frequency of HD sessions, fluid intake, urine volume, family history of dialysis, history of kidney transplantation, and medication prescriptions (for CKD-aP score and VAS score, in addition to the variables in Model 3, biochemical parameters were also adjusted) to determine the association between E-DII score as the independent variable and biochemical parameters, CKD-aP score, and VAS score as the dependent variables. Odds ratios (ORs) and 95% confidence intervals (CIs) were calculated to assess the risk of CKD-aP (dependent variables) according to the percentile of E-DII score (independent variable) using binary logistic regression in three models: Model 1 without adjustment; Model 2 with adjustment for age, sex, BMI, physical activity, diabetes, hypertension, smoking, job, marital status, education, income status, and energy intake; and Model 3 with adjustment for model 2 in addition to inter-dialysis weight gain, dialysis vintage, dialysis time, frequency of HD sessions, fluid intake, urine volume, family history of dialysis, history of kidney transplantation, medication prescriptions, and biochemical parameters.

## Results

The demographic characteristics of HD patients across the quartiles of E-DII are presented in Table 1. The E-DII scores of these participants ranged from − 4.13 (indicating a highly anti-inflammatory diet) to 4.63 (indicating a highly pro-inflammatory diet). The mean E-DII score in this study was 0.00 ± 2.23. The E-DII values were divided into four quartiles: quartile 1 ( < − 1.2960), quartile 2 (− 1.2960 to -0.1220), quartile 3 (− 0.1220 to 1.2388), and quartile 4 (> 1.2388). In comparison to quartile 4, participants in E-DII quartile 1 exhibited higher levels of physical activity, as evidenced by their education (*P* =.02), and received a higher amount of activated vitamin D prescription (*P* =.03). There were no other significant differences across categories of the E-DII score.

**Table 1:** The characteristics at baseline across quartiles of E-DII score in Hemodialysis patients

Characteristics, mean	Total	E-DII quartiles				*P* value
(SD) or *N* (%)	(*N* = 200)	Q1(*N* = 50)	Q2(*N* = 50)	Q3(*N* = 48)	Q4(*N* = 52)	
E-DII score	0.00 ± 2.23	-2.24 ± 0.77	-0.68 ± 0.34^a^	0.52 ± 0.42^b, d^	2.32 ± 0.95^c, e,f^	< 0.001
Age, y	55.54 ± 15.19	54.54 ± 14.59	54.04 ± 13.11	55.25 ± 15.88	58.23 ± 16.95	0.50
Sex, N (%)						0.50
Male	118(59)	30(60)	29(58)	32(67)	27(52)	
Female	82(41)	20(40)	21(42)	16(33)	25(48)	
Height, m	165.83 ± 11.74	166.22 ± 11.01	167.64 ± 10.29	167.13 ± 7.46	162.74 ± 15.93	0.14
Weight dry, kg	67.04 ± 14.62	66.17 ± 16.90	71.16 ± 13.88	66.46 ± 11.53	64.45 ± 15.08	0.11
Inter-Dialysis Weight Gain, kg	2.5 (1.5, 3.4)	2.5 (1.4, 3.5)	2.5 (1.5, 4,0)	2.5 (1.2, 3,0)	2 (1.2, 3,0)	0.60
BMI	24.73 ± 9.29	23.78 ± 4.90	25.30 ± 4.31	23.83 ± 4.02	25.92 ± 2.31	0.89
PA, met-min/wk	12 (5, 16.5)	15 (7.5, 20.4)	20 (7.5, 20.4)	6 (3, 12.2)	10 (4.5, 18.2)	0.20
Dialysis vintage, y	3.0 (1.3, 6.6)	3.0 (1.0, 7.6)	2.6 (1.0, 5.2)	3.4 (1.5, 7.0)	3.1 (1.5, 7.0)	0.62
Dialysis time, hr	3.95 ± 0.43	4.05 ± 0.54	3.96 ± 0.38	3.92 ± 0.48	3.89 ± 0.29	0.27
Frequency of hemodialysis sessions, times/wk	2.76 ± 0.68	2.76 ± 0.75	2.65 ± 0.56	2.76 ± 0.56	2.88 ± 0.81	0.42
Fluid intake, L	1 (0.7, 1.5)	1 (0.7, 1.63)	1.2 (0.7, 2.0)	1.2 (0.5, 1.5)	1 (0.7, 1.5)	0.19
Urine volume, L	0.1 (0, 0.5)	0.1 (0, 0.5)	0.1 (0, 0.5)	0.1 (0, 0.45)	0.1 (0, 0.5)	0.91
Diabetes (n, %)	77(38.5)	20(40)	19(38)	20(41.7)	18(34.6)	0.98
Hypertension (n, %)	94(47)	19(38)	23(46)	21(43.8)	31(59.6)	0.16
Family history of dialysis	42(21)	12(24)	12(24)	10(20.8)	8(15.4)	0.68
History of kidney transplantation	37(18.5)	7(14)	8(16)	10(20.8)	12(23.1)	0.62
Smoking (N) (%)	24(12)	4(8)	3(6)	13(27.1)	4(7.7)	0.26
Job, N (%)						0.06
Housekeeper	27(13.5)	8(16)	4(8)	8(16.7)	7(13.5)	
Retired	69(34.5)	17(34)	17(34)	12(25)	23(44.2)	
Employee	52(26)	12(24)	9(18)	16(33.3)	15(29.8)	
Self-employment	11(5.5)	2(4)	4(8)	4(8.3)	1(1.9)	
Others	39(20.5)	11(22)	16(28)	9(16.7)	6(11.5)	
Marital status, N (%)						0.24
Married	139(69.5)	38(76)	35(70)	29(60.4)	37(71.15)	
Single	33(16.5)	9(18)	6(12)	10(20.8)	8(15.4)	
Divorced	12(6)	2(4)	4(8)	4(8.3)	2(3.8)	
Dead spouse	16(8)	1(2)	5(10)	5(10.4)	5(9.6)	
Education, N (%)						0.02
Illiterate	104(52)	26(52)	21(42)	28(56)	29(58)	
Elementary diploma	50(25)	14(28)	12(24)	14(28)	10(20)	
Bachelor	9(4.5)	9(18)	9(18)	9(18)	9(18)	
Master of science	14(7)	2(4)	7(14)	1(2)	4(8)	
Income status, N (%)						0.25
< 5 million Rials	53(26.5)	9(18)	15(30)	11(20.9)	18(35)	
5–10 million Rials	85(42.5)	27(54)	16(32)	21(43.8)	21(41)	
10–20 million Rials	45(22.5)	9(18)	10(20)	14(29.2)	12(23)	
> 20 million Rials	17(8.5)	5(10)	9(18)	2(4.2)	1(2)	
Medication prescriptions						
Calcium carbonate 500 mg, time/day	1 (0, 3)	1 (0, 3)	1 (0, 3)	1 (0, 3)	1 (0, 2)	0.73
Sevelamer hydrochloride 800 mg, time/day	0.5 (0, 1)	0.5 (0, 1)	1 (0, 2)	0.5 (0, 1)	0.5 (0, 1)	0.28
Calcitriol 0.25 mcg, time/day	1 (0, 2)	0.1 (0, 1)	1 (0, 2)	0.1 (0,1.8)	0.5 (0,1)	0.01
Corticosteroids, N (%)	15(7.5)	4(8)	4(8)	3(6.3)	4(7.7)	0.22
Lipid-lowering drugs, N (%)	42(21)	8(16)	11(22)	11(22.9)	12(23.1)	0.98
Antihypertensive drugs, N (%)	94(47)	19(38)	23(46)	21(43.8)	31(59.6)	0.16

Continuous data with normal distribution are expressed as means ± SDs and continuous data with skewed distribution as median (IQR)

a Significant difference between quartiles 1 and 2; b Significant difference between quartiles 1 and 3; c Significant difference between quartiles 1 and 4; d Significant difference between quartiles 2 and 3; e Significant difference between quartiles 2 and 4; f Significant difference between quartiles 3 and 4;

∗ The significant difference was determined using ANOVA for normally distributed data, Kruskal-Wallis for non-normally distributed data, and Chi-square for categorical variables. (*P* < .05). Post hoc (LSD)ccording to the pattern

Abbreviations: PA, physical activity, wk, week; hr, hours

Regarding the dietary intake of participants based on E-DII quartiles, significant differences were observed in the intake of various nutrients, including, polyunsaturated fats, omega-3 fatty acids, omega-6 fatty acids, fiber, magnesium, zinc, selenium, Vitamin A, beta carotene, Vitamin E, Vitamin D, Vitamin C, riboflavin, B6, Folic acid and tea (*P* < .05), as tested by ANOVA (Table 2).

**Table 2:** The mean ± sd of nutrients and food intake across quartiles of E-DII in Hemodialysis patients

Characteristics, mean	Total	E-DII quartiles				*P*-value
(SD) or N (%)	(*N* = 200)	Q1(*N* = 50)	Q2(*N* = 50)	Q3(*N* = 48)	Q4(*N* = 52)	
Energy, kcal/d	2178.79 ± 840.31	2230.79 ± 1065.25	2314.55 ± 931.05	2019.88 ± 699.39	2144.92 ± 579.38	0.35
Carbohydrates intake, g	335.79 ± 131.03	334 ± 154.8	353.94 ± 145.64	308.32 ± 104.15	345.4 ± 111.62	0.34
Protein intake, g	75.92 ± 33.51	80.48 ± 42.43	82.42 ± 38.5	70.2 ± 27.04	70.56 ± 20.81	0.13
Total fat intake, g	62.56 ± 34.93	68.2 ± 40.93	67.32 ± 40.77	58.93 ± 30.55	55.9 ± 24.16	0.20
Saturated fats, g	18.49 ± 10.89	19.35 ± 11.61	18.55 ± 10.31	18.79 ± 11.34	17.34 ± 10.52	0.82
Cholesterol, mg	202 (136.04, 294.07)	179 (132.49, 253.09)	212 (135.22, 258.21)	218 (145.43, 322.21)	203 (125.73, 321.21)	0.56
Trans fats, g	0.33 (0.2, 0.52)	0.29 (0.21, 0.42)	0.32 (0.21, 0.48)	0.39 (0.18, 0.56)^b, d^	0.31 (0.18, 0.57)^c, e^	0.87
Monounsaturated fats, g	20.11 ± 12.33	21.82 ± 13.71	21.8 ± 14.81	18.56 ± 10.84	18.28 ± 9.05	0.28
Polyunsaturated fats, g	17 (11.55, 25.25)	16 (10.06, 23.51)	14 (9.02, 21.79)	12 (8.73, 15.63)	12 (7.62, 16.95)	0.03
Omega 3 fatty acids, g	0.30 (0.17, 0.55)	0.48 (0.32, 0.71)	0.43 (0.23, 0.61)	0.39 (0.18, 0.56)	0.17 (0.09, 0.26)	< 0.001
Omega 6 fatty acids, g	13.32 ± 9.48	15.31 ± 10.41	15.48 ± 12.8	11.62 ± 6.82^b^	10.9 ± 5.44^c, e^	0.02
Fiber, g	18.45 ± 9.78	23.33 ± 13.09	20.18 ± 9.67	15.7 ± 6.42^b, d^	14.62 ± 5.^73c, e^	< 0.001
Magnesium, mg	220.38 ± 115.51	274.76 ± 136.96	252.01 ± 128.53	187.75 ± 74.06^b, d^	167.79 ± 73.57^c, e^	< 0.001
Iron, mg	15 (12.21, 20.19)	16 (12.41, 23.8)	15 (12.08, 25.11)	14 (11.7, 17.97)	15 (12.08, 19.18)	0.24
Zinc, mg	5 (3.93, 7.07)	6 (4.51, 8.72)	6 (3.73, 8.84)	5 (4.11, 6.74)	4 (3.46, 5.89)	0.004
Selenium, µg	0.03 (0.01, 0.36)	0.13 (0.01, 0.56)	0.06 (0.01, 0.4)	0.12 (0.01, 0.44)	0.01 (0, 0.03)	< 0.001
Vitamin A, RE	633 (444.4, 1004.79)	854 (570.98, 1319.88)	840 (482.27, 1166.31)	539 (455.5, 924.65)	437 (315.88, 650.55)	< 0.001
Beta carotene	472 (268.08, 956.92)	990 (679.99, 1663.46)	555 (367.21, 1338.55)	430 (268.08, 645.35)	255 (117.24, 388.08)	< 0.001
Vitamin E, mg	7 (3.88, 12.1)	12 (7.82, 16.36)	9 (5, 13.53)	5 (3.85, 9.73)	4 (1.97, 6.62)	< 0.001
Vitamin D, µg	0.93 (0.47, 1.68)	1.34 (0.9, 2.06)	1.05 (0.6, 1.93)	0.67 (0.33, 1.55)	0.57 (0.18, 1)	< 0.001
Vitamin C, mg	88 (58.18, 139.46)	132 (87.77, 180.43)	106 (73.75, 175.99)	72 (47.54,107.48)	72 (42.47, 97.79)	< 0.001
Thiamin, mg	2.14 ± 0.81	2.16 ± 0.94	2.24 ± 0.9	1.96 ± 0.63	2.19 ± 0.73	0.35
Riboflavin, mg	1.4 ± 0.72	1.57 ± 0.83	1.52 ± 0.77	1.28 ± 0.63^b^	1.21 ± 0.57^c, e^	0.02
Niacin, mg	22.26 ± 9.4	22.54 ± 11.96	24.04 ± 11.14	20.8 ± 7.23	21.61 ± 5.83	0.35
B6, mg	0.32 (0.21, 0.56)	0.47 (0.28, 0.87)	0.36 (0.24, 0.58)	0.3 (0.21, 0.4)	0.25 (0.14, 0.43)	< 0.001
Folic acid, µg	208 (156.13, 279.86)	251 (189.79, 354.9)	236 (162.8, 389.49)	204 (147.64, 248.01)	167 (122.97, 215.29)	< 0.001
Vitamin B12, µg	1.8 (1.19, 2.99)	1.79 (1.26, 3.64)	1.7 (1.19, 3.08)	1.92 (1.46, 2.86)	1.67 (1.07, 2.32)	0.36
Caffeine, mg	0.17 (0.07, 0.63)	0.09 (0.03, 0.26)	0.41 (0.14, 0.8)	0.32 (0.1, 0.9)	0.16 (0.07, 0.35)	0.49
Onion, g	4.75 (0, 9.5)	4.75 (0, 9.5)	4.75 (0.97, 9.5)	2.89 (0, 8.31)	1.11 (0, 4.75)	0.14
Tea, g	2 (0.86, 4)	1 (0.29, 2)	2 (0.96, 4)	2 (1.14, 4)	2 (1.04, 4)	0.05
Pepper, g	0.88 (0.19, 3.31)	0.81 (0.14, 3.4)	0.77 (0.24, 3.28)	1.24 (0.77, 3.28)	0.9 (0.02, 3.38)	0.97
Garlic, g	0.11 (0, 0.38)	0.11 (0.02, 0.35)	0.07 (0, 0.32)	0.15 (0, 0.59)	0.1 (0, 0.4)	0.80

Continuous data with normal distribution are expressed as means ± SDs and continuous data with skewed distribution as median (IQR)

a Significant difference between quartiles 1 and 2; b Significant difference between quartiles 1 and 3; c Significant difference between quartiles 1 and 4; d Significant difference between quartiles 2 and 3; e Significant difference between quartiles 2 and 4; f Significant difference between quartiles 3 and 4;

∗ The significant difference was determined using ANOVA for normally distributed data, and Kruskal-Wallis for non-normally distributed data. (*P* < .05). Post hoc (LSD)ccording to the pattern

Table 3 presents the mean ± SD or median (IQR) of biochemical parameters and the prevalence rate of CKD-aP across the quartiles of E-DII. No significant differences were observed in the biochemical parameters between the E-DII quartiles (*P* ≥.05). The prevalence rate of CKD-aP in this study was 40% (*n* = 80). We observed a significant increase in the rate of patients with CKD-aP across the E-DII quartiles (*P* < .05). Additionally, there was a significant increase in the CKD-aP score, VAS score, pruritus during the last month, frequent pruritus, daily activities, mood and behavior, ability to concentrate, sexual desire, and severity of CKD-aP across categories of the E-DII score (*P* < .05).

**Table 3:** The mean ± sd of biochemical parameter and chronic kidney disease-associated pruritus across quartiles of E-DII in Hemodialysis patients

Characteristics, mean	Total	E-DII quartiles				*P*-value
,SD or *N* ,%	(*N* = 200)					
		Q1(*N* = 50)	Q2(*N* = 50)	Q3(*N* = 48)	Q4(*N* = 52)	
FBS, mg/dL	110.22 ± 46.19	113.6 ± 41.93	113.1 ± 64.72	110.37 ± 34.91	104.06 ± 37.82	0.71
HB, g/dL	12 (10.7, 12.9)	11 (10.55, 12.9)	12 (10.15, 13.18)	12 (10.83, 12.48)	12 (11.03, 13)	0.51
Iron, mg/dL	58 (39.25, 80)	61 (46.5, 84.25)	58 (43.75, 84.25)	57 (35, 79)	56 (37.5, 75.44)	0.73
Ferritin, ng/mL	266 (122, 448.8)	292 (154, 437)	222 (53.43, 436)	235 (130, 485.73)	314 (94.5, 456.45)	0.54
Serum total iron binding capacity, µg/dL	236.6 ± 71.1	240.97 ± 81.16	247.46 ± 54.42	216.23 ± 63.52	240.75 ± 79.32	0.14
Hematocrit, %	36 (33.0, 39.0)	34 (31.8, 37.33)	37 (32.75, 39.48)	36 (32.4, 39.80)	37 (32.8, 39.70)	0.06
Platelets, 10*3/µg	182 (143.5, 226.75)	170 (147.25, 216.25)	195 (125.25, 244)	202 (145.25, 250)	178 (143, 219.5)	0.41
Creatinine, mg/dL	7.19 ± 4.16	6.76 ± 2.18	8.47 ± 7.01	6.51 ± 2.35	7 ± 2.89	0.08
Calcium, mg/dL	8.52 ± 0.85	8.42 ± 0.8	8.39 ± 0.9	8.49 ± 0.87	8.78 ± 0.78	0.07
Sodium, mmol/L	137.75 ± 15.96	137.45 ± 18.19	138.21 ± 18.65	138.58 ± 3.58	136.84 ± 18.1	0.95
Potassium, mmol/L	5.34 ± 0.86	5.45 ± 0.89	5.44 ± 1.01	5.25 ± 0.79	5.21 ± 0.74	0.34
Phosphate, mg/dL	4.87 ± 1.22	4.82 ± 1.3	5.22 ± 1.18	4.82 ± 1.32	4.62 ± 1.03	0.10
Ca x P, mg2/dL2	41.45 ± 10.62	40.22 ± 10.4	43.93 ± 9.69	41 ± 12.1	40.73 ± 10.14	
Albumin, g/ dL	4.0 (3.7, 4.3)	4.0 (3.7, 4.39)	4.0 (3.58, 4.2)	3.99 (3.53, 4.2)	4.1 (3.89, 4.35)	0.30
iPTH, pg/mL	476 (218.25, 833)	574 (343.6, 850.25)	569 (282.75, 948.1)	386 (189.75, 836.5)	385 (142.5, 643)	0.06
Pre-dialysis BUN, mg/dL	49 (37, 58)	50 (36, 56)	52 (41.75, 62)	50 (35.5, 64.75)	47 (38.25, 53.75)	0.40
Post-dialysis BUN, mg/dL	17 (12, 21)	15 (11, 22)	18 (16, 23)	18 (13, 20)	15 (12, 17)	0.10
Urea reduction ratio	0.66 (0.6, 0.73)	0.66 (0.6, 0.76)	0.64 (0.59, 0.69)	0.64 (0.6, 0.73)	0.68 (0.62, 0.74)	0.10
Kt/V	1.26 (1.1, 1.5)	1.24 (1.09, 1.66)	1.25 (1.06, 1.39)	1.2 (1.05, 1.41)	1.33 (1.16, 1.59)	0.07
CKD-aP, N (%)	80(40)	11(22)	22(44)	23(47.9)	24(46.2)	0.02
CKD-aP Score	5 (4,6.75)	4 (1,5)	5 (4,7)	6 (4.25,6)	6 (4.5,7)	0.04
Pruritus during last 1 months N (%)	73(36.5)	7(14)	22(44)	21(43.75)	24(42.2)	0.002
Pruritus during last 5 months N (%)	64(32)	7(14)	18(36)	17(35.41)	22(42.3)	0.20
Frequent pruritus N (%)						0.03
Daily	38(19)	8(16)	9(18)	9(18.8)	12(23.1)	
Weekly	28(14)	4(8)	9(18)	9(18.8)	6(11.5)	
Fortnightly	8 (4)	1(2)	2(4)	2(4.2)	3(5.8)	
Monthly	6 (3)	0(0)	4(8)	2(4.2)	0(00)	
Time N (%)						0.11
> 30 min	38(19)	4(8)	12(24)	11(22.9)	11(21.2)	
30 min	10(5)	2(4)	1(2)	3(6.3)	4(7.7)	
60 min	8(4)	0(0)	3(6)	4(8.3)	1(1.9)	
< 60 min	22(11)	4(8)	6(12)	5(10.4)	7(13.5)	
Effect of CKD-aP on						
sleep N (%)	43(21.5)	6(12)	11(22)	11(22)	16(32)	0.57
Daily activities N (%)s	20(10)	3(6)	6(12)a	3(6.25)	8(15.4)	0.006
Mood and behavior N (%)	29(14.5)	3(6)	10(20)	5(10.4)	11(21.2)	0.02
ability to concentrate N (%)	27(13.5)	3(6)	10(20)	5(10.4)	9(17.3)	0.01
Sexual desire N (%)	10(5)	2(4)	3(6)	3(6.3)	2(3.8)	0.03
VAS score	1.5 (0, 5)	1 (1, 4)	2 (1, 5)	3 (1, 5)	4 (2, 5)	0.02
Severity CKD-aP N (%)						0.01
Mild	22(11)	8(16)	7(14)	5(10.4)	2(3.8)	
Moderate	41(20.5)	7(14)	13(26)	14(29.2)	7(13.5)	
Severe	17(8.5)	1(2)	7(14)	1(2.1)	8(15.4)	

Continuous data with normal distribution are expressed as means ± SDs and continuous data with skewed distribution as median (IQR)

a Significant difference between quartiles 1 and 2; b Significant difference between quartiles 1 and 3; c Significant difference between quartiles 1 and 4; d Significant difference between quartiles 2 and 3; e Significant difference between quartiles 2 and 4; f Significant difference between quartiles 3 and 4;

∗ The significant difference was determined using ANOVA for normally distributed data, Kruskal-Wallis for non-normally distributed data, and Chi-square for categorical variables. (*P* < .05). Post hoc (LSD)ccording to the pattern

Abbreviations: FBS, fasting blood sugar, BUN, blood urea nitrogen; Ca x P, absolute value of calcium concentration multiplied by absolute value of phosphate concentration; iPTH, intact parathyroid hormone; Chronic kidney disease-associated pruritus (CKD-aP); VAS, Visual Analogue Scale

Based on the generalized linear models analyses presented in Table 4, the results were adjusted for various covariates across three models. Table 4 presents the association between the EII score (as an independent variable), and CKD-aP and VAS scores (as dependent variables). In model 3, inverse and positive statistically significant association (*P* ≤.05) was verified between E-DII and CKD-aP scores and VAS score, respectively.

**Table 4:** Generalized linear models of the association chronic kidney disease-associated pruritus and E-DII in Hemodialysis patients

		Q1		Q2		Q3		Q4		*p*-Trend
		β (SE)	*p*	β (SE)	*p*	β (SE)	*p*	β (SE)	*p*	
**CKD-aP**										
CKD-aP Score	Model 1	Ref.	-	0.78 (0.51)	0.12	0.63 (0.54)	0.24	0.82 (0.57)	0.15	0.24
	Model 2	Ref.	-	1.23 (0.47)	0.03	1.05 (0.51)	0.33	1.30 (0.49)	0.54	0.94
	Model 3	Ref.	-	3.59 (0.30)	< 0.001	2.03 (0.19)	< 0.001	2.84 (0.28)	< 0.001	0.004
VAS Score	Model 1	Ref.	-	1.56 (0.92)	0.08	1.08 (0.97)	0.26	1.52 (1.01)	0.13	0.26
	Model 2	Ref.	-	1.33 (0.88)	0.13	1.38 (0.93)	0.13	1.33 (0.90)	0.11	0.17
	Model 3	Ref.	-	1.23 (0.78)	0.73	1.34 (0.69)	0.26	1.44 (0.95)	< 0.001	< 0.001

Model 1: Calculated using generalized linear models, E-DII quartiles entered as an independent variable. In this test, bold values indicate the significance level below 0.05 is defined (*p* < .05)

Model 2: Calculated using generalized linear models, E-DII quartiles entered as an independent variable, and adjusted for the effect of age, sex, BMI, physical activity, diabetes, hypertension, smoking, job, marital status, education, income status, and energy intake

Model 3: Calculated using generalized linear models, E-DII quartiles entered as an independent variable, and adjusted for the effect of age, sex, BMI, physical activity, diabetes, hypertension, smoking, job, marital status, education, income status, and energy intake, inter-dialysis weight gain, dialysis vintage, dialysis time, frequency of hemodialysis sessions, fluid intake, urine volume, family history of dialysis, history of kidney transplantation, medication prescriptions, and biochemical parameters

Table 5 shows the odds ratios (ORs) and 95% confidence intervals (CIs) for the risk of CKD-aP (dependent variables) according to the E-DII score (independent variable) in HD patients. We found a positive association between the incidence of CKD-aP and the E-DII score in model 1 (OR = 1.19, 95% CI = 1.02–1.40), model 2 (OR = 1.29, 95% CI = 1.00-1.65), and model 3 (OR = 1.41, 95% CI = 1.00-1.98).

**Table 5:** – Odds ratio (95% CI) for risk of chronic kidney disease-associate pruritus (dependent variables) according to the E-DII score (independent variable) in Hemodialysis patients

Variable	OR (CI)	B	*P* value
Model 1	1.19(1.02–1.40)	0.18	0.02
Model 2	1.29(1.01–1.65)	0.25	0.04
Model 3	1.41(1.01–1.98)	0.34	0.04

*P* < .05 statistically significant by multivariable logistic regression

Model 1. binary logistic regression analysis without adjustment

Model 2. binary logistic regression analysis with age, sex, BMI, physical activity, diabetes, hypertension, smoking, job, marital status, education, income status, and energy intake

Model 3. linear regression analysis with adjustment for model 2 in addition to inter-dialysis weight gain, dialysis vintage, dialysis time, frequency of hemodialysis sessions, fluid intake, urine volume, family history of dialysis, history of kidney transplantation, and medication prescriptions

## Discussion

To the best of our knowledge, this is the first cross-sectional study to investigate the association between E-DII and CKD-aP in patients undergoing HD. Overall, our findings support the hypothesis that a pro-inflammatory diet may contribute to an increased risk of CKD-aP. According to these findings, compared to quartile 4, individuals in E-DII quartile 1 had lower CKD-aP scores and VAS scores. The linear regression analysis demonstrated a significant association between the E-DII score and CKD-aP scores, as well as VAS scores. The association of the E-DII score with CKD-aP score, and VAS score persisted and even strengthened after adjusting for various factors. Additionally, the logistic regression analysis revealed that a pro-inflammatory diet may increase the risk of CKD-aP.

The CKD-aP is a common and distressing symptom observed in chronic HD patients. Previous studies have reported varying prevalence rates of CKD-aP among HD patients. These differences can be attributed to differences in measurement methods; for example, some studies estimated the current prevalence of CKD-aP while others estimated the lifetime prevalence. In our study, we evaluated the current prevalence of CKD-aP. The prevalence rate of CKD-aP in this study was 40%. Our findings align with similar studies conducted in Iran, such as those by Malekmakan et al. and Akhyani et al., which reported CKD-aP prevalence rates of 40.2% and 41.9%, respectively [26, 27]. Another study by Ramezanzade et al. found that 54.01% of patients complained of CKD-aP [28].

Several studies have proposed that inflammatory conditions, including elevated levels of pro-inflammatory cytokines, may play a role in the development of CKD-aP [29]. Several studies have provided evidence of the influence of diet on inflammation and the relationship between specific dietary components and varying levels of inflammation [30]. The Dietary Inflammatory Index (DII) is a valuable tool for assessing the inflammatory potential of the diet and can be applied to populations with available dietary data [12, 13]. Furthermore, it can serve as a valuable tool for assessing and guiding individuals in decreasing inflammation levels and potentially reducing the risk of certain chronic diseases [12]. Dietary compounds with antioxidant and anti-inflammatory properties are crucial in reducing inflammation and cardiovascular risk [31]. Several research has demonstrated the effectiveness of dietary interventions in mitigating chronic inflammation [12]. In individuals with chronic kidney disease (CKD), various potential anti-oxidative and anti-inflammatory agents, such as vitamin C, vitamin E, fish oil, vitamin A/carotenoids, and carnitine, have been shown to decrease inflammation [32]. Moreover, interventions involving flaxseed oil [33], soy isoflavones [34], selenium [35], and omega-3 fatty acids [36] have demonstrated a reduction in inflammation among HD patients. Conversely, the consumption of saturated fats, high glycemic index foods, and foods with a high omega-6/omega-3 ratio has been associated with increased levels of inflammation [37]. Consistent with these findings, our study revealed that intakes of polyunsaturated fats, omega-3 fatty acids, and omega-6 fatty acids were higher in quartile 1, which represents a more anti-inflammatory diet. In addition to the aforementioned, several studies have investigated the association between DII and inflammation in HD patients. In a cross-sectional study of 109 HD patients, Kizil et al. demonstrated that an increase in the DII category was associated with higher CRP levels [19]. Similarly, another cross-sectional study with 160 HD patients reported a significant positive association between the DII score and CRP levels [18]. Other studies conducted among different populations have also shown significant associations between DII and inflammatory biomarkers effective in CKD-aP, such as interleukin-6, interleukin-2, and tumor necrosis factor-α [38]. As previously mentioned, elevated levels of inflammatory biomarkers can contribute to an increased risk and severity of CKD-aP in HD patients. Therefore, higher consumption of an anti-inflammatory diet can potentially reduce the risk and severity of CKD-aP by decreasing inflammatory biomarkers. Consistent with our study, Tseng et al. found that vegetarian patients had lower levels of inflammatory factors such as hs-CRP and IL-2, as well as lower visual-simulation scores (Analog Score and VAS) and CKD-aP scores compared to non-vegetarian patients. This study suggests that a vegetarian diet, as an anti-inflammatory diet, may improve CKD-aP severity in HD patients [39].

It is noteworthy that serum albumin is considered a potential marker of systemic inflammation [40]; however, our findings showed no significant difference in albumin levels across E-DII categories. This may be explained by the fact that serum albumin is influenced by multiple factors other than inflammation, including nutritional intake, fluid status, protein losses during dialysis, comorbid conditions such as liver disease, and the presence of protein-energy wasting [41–43]. Additionally, given that protein intake did not significantly differ among participants, it is plausible that this nutritional stability contributed to similar albumin levels. Notably, studies have demonstrated that higher dietary inflammatory index (DII) scores resulted in elevated CRP levels, while no significant changes in serum albumin concentrations were observed in hemodialysis patients [18, 19].

In our study, no significant associations were observed between E-DII and various biochemical parameters, including HB, ALB, Cr, Urea, Ca, Na+, K+, and P, which was similar to the findings of Zeng et al. and Kizil et al. [18, 19].

While our study has some limitations, including its cross-sectional design and small sample size, which preclude determining causal relationships, another important limitation is the absence of data on HD membrane type and heparin use (LMWH vs. HMWH), as well as the lack of inflammatory marker data such as IL-6, CRP, IL-2, and other cytokines. Although we assessed the association between energy-adjusted dietary inflammatory index (E-DII) and CKD-associated pruritus (CKD-aP), it is possible that E-DII reflects factors beyond inflammation. Therefore, inclusion of these variables as potential confounding factors in future multivariate analyses would help clarify the mechanisms linking diet and pruritus in HD patients. However, despite these limitations, there are notable strengths that should be acknowledged. This study is the first cross-sectional investigation of the association between E-DII and CKD-aP in HD patients. The use of a validated Food Frequency Questionnaire (FFQ) for dietary assessment, along with rigorous statistical analyses such as linear regression and multivariable logistic regression in three different models while adjusting for various factors, provides a robust foundation for interpreting the data.

## Conclusion

In this cross-sectional study, it was found that HD patients who consumed a pro-inflammatory diet were associated with a higher incidence of CKD-aP. Future studies with prospective and interventional designs are required to clarify the association between this dietary index and CKD-aP among HD patients.

## Data Availability

The datasets generated in the current study are available from the corresponding author upon reasonable request.
